# Prognostic value of an integrated immune-inflammatory phenotype in surgically treated cervical cancer: survival modeling and immunohistochemical validation

**DOI:** 10.3389/fimmu.2026.1850891

**Published:** 2026-06-16

**Authors:** Lin Ran, Zhaoan Lian, Yong Tian, Li Qin, Yingchun Xiang, Xiaohao Yan, Chengyu Shui

**Affiliations:** 1 Department of Obstetrics and Gynecology, Central Hospital of Enshi Tujia and Miao Autonomous Prefecture, Enshi Clinical College of Wuhan University, Enshi, China; 2 Chengdu Huake Biology Research Center, Chengdu, China

**Keywords:** cervical cancer, immunohistochemistry, integrated immune-inflammatory phenotype, LASSO-COX, random survival forest, recurrence-free survival, systemic immune-inflammation index, tumor-infiltrating lymphocytes

## Abstract

**Background:**

Postoperative recurrence risk in cervical cancer remains heterogeneous, and conventional clinicopathological factors may not fully capture the contribution of the immune microenvironment and systemic inflammation. We investigated whether an integrated immune-inflammatory phenotype combining stromal tumor-infiltrating lymphocytes (TILs) and the systemic immune-inflammation index (SII) could improve recurrence-free survival (RFS) stratification after surgery.

**Methods:**

This retrospective cohort study included 612 patients with cervical cancer who underwent primary surgery between January 2020 and December 2025. Stromal TILs were assessed on hematoxylin-eosin sections, and pretreatment SII was calculated from blood counts. An integrated immune-inflammatory phenotype was defined as favorable, poor, or intermediate. Kaplan-Meier analysis, restricted cubic spline modeling, and multivariable Cox regression were performed. Prognostic performance was compared across a clinical Cox model, an immune-extended Cox model, LASSO-Cox, CoxBoost, and random survival forest (RSF) using C-index, time-dependent area under the curve (AUC), integrated Brier score (IBS), calibration, and 36-month landmark decision curve analysis. Tissue-level validation was performed using immunohistochemical assessment of CD8, CD163, and PD-L1.

**Results:**

During follow-up, 119 patients experienced an RFS event. Kaplan-Meier analysis showed significant RFS differences according to TIL category, SII category, and integrated phenotype. Restricted cubic spline analysis demonstrated a significant overall association between SII and recurrence risk, without marked nonlinearity. In multivariable analysis, FIGO IIIC disease, positive margin status, and the poor integrated phenotype remained independently associated with worse RFS. In model comparison, the immune-extended Cox model had the lowest IBS (0.278), LASSO-Cox achieved the highest C-index (0.782) and the best discrimination at 24 and 36 months, and RSF showed the highest 60-month AUC. In 36-month decision curve analysis, LASSO-Cox and RSF showed the greatest net benefit at intermediate thresholds. Immunohistochemical validation showed that the favorable phenotype was characterized by higher CD8+ cell density, lower CD163+ cell density, a higher CD8/CD163 ratio, and higher PD-L1 combined positive score.

**Conclusion:**

An integrated immune-inflammatory phenotype combining stromal TILs and SII was independently associated with postoperative recurrence risk in cervical cancer and corresponded to distinct tissue immune states. This phenotype may provide a practical framework for recurrence risk stratification, while LASSO-Cox and RSF offer complementary prognostic perspectives.

## 1 Introduction

Cervical cancer remains a major global health problem despite substantial progress in vaccination, screening, and treatment. According to the most recent GLOBOCAN estimates, cervical cancer accounted for 662,301 new cases and 348,874 deaths worldwide in 2022, and it remains one of the leading causes of cancer death among women in many low- and middle-income settings (1). The World Health Organization has framed cervical cancer elimination as a realistic public health goal, but that ambition depends not only on prevention and early detection, but also on improving risk assessment and treatment for women who already have invasive disease (2).

For patients who undergo primary surgery, postoperative outcome is still heterogeneous. Even among women with apparently similar stage and pathological findings, recurrence risk is not uniform, and relapse after initial treatment remains clinically important (3). Conventional prognostic assessment still relies mainly on established clinicopathological factors such as stage, tumor size, depth of stromal invasion, lymphovascular space invasion, nodal status, and margin status (4). These variables are indispensable, but they do not fully capture the biological diversity of cervical cancer, particularly the contribution of the host immune response and the tumor microenvironment.

This gap is especially relevant in cervical cancer because the disease develops in the setting of persistent HPV-driven carcinogenesis and a highly dynamic immune microenvironment. Increasing evidence suggests that local immune cell composition is closely linked to tumor behavior and treatment response (5, 6). Stromal tumor-infiltrating lymphocytes (TILs), in particular, have attracted attention as a practical histology-based marker. Recent work suggests that stromal TILs may carry stronger prognostic information than intraepithelial TILs in cervical cancer, and CD8-positive lymphocyte infiltration has also been associated with more favorable outcome in patients treated with radiotherapy or chemoradiotherapy (6–8). These observations support the idea that local immune contexture is clinically meaningful, but they also raise a broader question: whether tissue-level immune infiltration alone is sufficient for risk stratification in routine practice.

At the same time, systemic inflammatory markers have emerged as accessible indicators of host-tumor interaction. Among them, the systemic immune-inflammation index (SII), derived from peripheral blood cell counts, has shown prognostic value across several solid tumors, including cervical cancer (9). Recent pooled analyses suggest that elevated pretreatment SII is associated with worse survival outcomes in cervical cancer (10, 11). The appeal of SII is obvious: it is inexpensive, routinely available, and easy to standardize. Still, it reflects systemic inflammatory status rather than the local immune architecture within the tumor. For that reason, it is unlikely to be a complete surrogate for the tumor immune microenvironment.

Taken together, these findings suggest that local immune infiltration and systemic inflammation may provide complementary rather than redundant information. Yet most published studies in cervical cancer have evaluated these dimensions separately. Research has usually focused on stromal TILs, specific lymphocyte subsets, checkpoint markers, or blood-based inflammatory indices one at a time (6, 7, 9, 10). Far fewer studies have attempted to combine a practical tissue-derived immune measure with a routine systemic inflammatory marker into a clinically usable phenotype, and fewer still have examined whether such a phenotype corresponds to distinct tissue immune states. This is an important unmet need, because a composite framework may better reflect the balance between antitumor immunity and protumor inflammation than either dimension alone.

A related issue is model construction. Interest in machine learning for cervical cancer prognosis has grown rapidly, and recent reviews indicate that machine learning methods can improve survival prediction beyond traditional approaches in selected datasets (12). However, many published models are either weakly interpretable or heavily dependent on variables that are not readily translatable to routine postoperative practice. For clinical use, predictive performance is not enough. A useful model should also preserve interpretability, align with known biology, and ideally be supported by orthogonal evidence from tissue analysis.

On that background, we designed the present study to address three linked questions. First, can a simple integrated immune-inflammatory phenotype, derived from stromal TILs and SII, improve recurrence-free survival stratification in surgically treated cervical cancer? Second, how does this phenotype perform when tested across conventional survival modeling, regularized regression, and machine learning approaches? Third, does the phenotype correspond to biologically distinct immune states at the tissue level? To address these questions, we combined conventional survival analysis, restricted cubic spline modeling, multivariable Cox regression, model comparison using LASSO-Cox, CoxBoost, and random survival forest, decision curve analysis, and immunohistochemical validation with CD8, CD163, and PD-L1. The main novelty of this work lies not in proposing another isolated biomarker, but in linking a pragmatic integrated phenotype to prognostic modeling and tissue-level immune validation in the same cervical cancer cohort.

## 2 Materials and methods

### 2.1 Study design and patients

This was a retrospective single-center cohort study of patients with primary cervical cancer who underwent surgical treatment between January 2020 and December 2025. The analytic cohort was restricted to patients with available preoperative clinicopathological data, pretreatment blood counts, assessable hematoxylin-eosin sections for stromal TIL evaluation, and follow-up information sufficient for recurrence-free survival (RFS) analysis. Patients with missing data in the prespecified variables used for survival modeling were excluded from the final analytic dataset. Reporting of the prognostic analyses followed the general principles of the REMARK recommendations where applicable (13).

This study was conducted in accordance with the Declaration of Helsinki. The study was approved by the Ethics Committee of Enshi Tujia & Miao Autonomous Prefecture Central Hospital (Approval No.: 2025039). Given the retrospective nature of the study and the use of anonymized/de-identified clinical data, the requirement for informed consent was waived by the committee.

### 2.2 Clinicopathological variables and outcome definition

The following clinicopathological variables were collected from the medical record and pathology reports: age, body mass index (BMI), menopausal status, histology, tumor grade, 2018 FIGO stage, tumor size, stromal invasion, lymphovascular space invasion (LVSI), parametrial invasion, margin status, surgical approach, and adjuvant treatment. Stage assignment followed the 2018 FIGO staging system for cervical cancer (14). For modeling purposes, FIGO stage was grouped as IA, IB, IIA, and IIIC. Lymph node status was described descriptively, but was not entered simultaneously with FIGO group into the core prognostic models because nodal involvement is incorporated into the 2018 FIGO IIIC category.

The primary endpoint was RFS, defined as the interval from the date of surgery to the date of first documented recurrence. Patients without recurrence were censored at the date of last follow-up.

### 2.3 Assessment of stromal TILs, SII, and the integrated immune-inflammatory phenotype

Pretreatment SII was derived from routine peripheral blood counts using the standard formula: platelet count × neutrophil count/lymphocyte count. Because the SII distribution was right-skewed, log-transformed SII was used in regression and machine-learning analyses. For spline analysis, SII was winsorized at the 99th percentile to reduce the influence of extreme values.

Stromal TILs were assessed on hematoxylin-eosin stained sections as the percentage of stromal area within the invasive tumor occupied by mononuclear inflammatory cells, following published recommendations for morphological TIL assessment, with emphasis on the stromal rather than intraepithelial compartment (15). In line with the predefined analysis plan, stromal TILs were dichotomized at 20% for survival stratification. SII was dichotomized at the cohort median for categorical analyses. An integrated immune-inflammatory phenotype was then defined as follows: favorable, high stromal TILs combined with low SII; poor, low stromal TILs combined with high SII; and intermediate, all remaining combinations.

### 2.4 Immunohistochemical validation

To examine whether the integrated immune-inflammatory phenotype corresponded to distinct tissue immune states, an immunohistochemical validation analysis was performed in representative cases from the favorable and poor phenotype groups with adequate archived formalin-fixed paraffin-embedded tissue and clearly assessable invasive tumor areas. To maintain balanced comparison between groups, 30 cases were included from each phenotype group. Formalin-fixed, paraffin-embedded tumor sections were stained for CD8, CD163, and PD-L1 according to routine laboratory procedures. CD8-positive and CD163-positive immune cell densities were recorded as cells/mm^2^, and the CD8/CD163 ratio was calculated. PD-L1 expression was evaluated using the combined positive score (CPS), defined as the number of PD-L1-staining tumor cells, lymphocytes, and macrophages divided by the total number of viable tumor cells, multiplied by 100.

To further examine the relationship between pretreatment systemic inflammation and local immune context, we performed exploratory analyses in the immunohistochemical validation subset by linking preoperative SII to tissue immune markers. Spearman correlation analyses were used to assess the association of log-transformed SII with CD8+ cell density, CD163+ cell density, CD8/CD163 ratio, and PD-L1 combined positive score (CPS). Exploratory linear regression models were then fitted for each tissue marker with adjustment for FIGO group and tumor size.

### 2.5 Conventional survival analysis

Baseline characteristics were summarized as median (Q1, Q3) for continuous variables and n (%) for categorical variables. Comparisons between recurrence groups were performed using the Wilcoxon rank-sum test for continuous variables and the Pearson chi-squared test or Fisher’s exact test for categorical variables, as appropriate. Comparisons across integrated immune-inflammatory phenotype groups were performed using the Kruskal-Wallis test for continuous variables and the Pearson chi-squared test or Fisher’s exact test for categorical variables.

RFS was estimated using the Kaplan-Meier method, and differences between strata were compared using the log-rank test. To examine the shape of the association between SII and recurrence risk, restricted cubic spline analysis was performed within a Cox proportional hazards model using winsorized SII and four knots. The overall association and the nonlinear component were evaluated from the spline model.

### 2.6 Multivariable Cox regression

Multivariable Cox proportional hazards regression was used to estimate adjusted hazard ratios (HRs) and 95% confidence intervals (CIs) for RFS. The core multivariable model included age, BMI, histology, grade, FIGO group, LVSI, margin status, adjuvant treatment, and integrated immune-inflammatory phenotype. These variables were prespecified on clinical grounds and retained to preserve interpretability of the main prognostic model. Proportional hazards assumptions were checked using scaled Schoenfeld residuals.

### 2.7 Model development and internal validation

To compare conventional and machine-learning survival models, the cohort was split into a training set and a test set in a 7:3 ratio using stratified random sampling by RFS event status. Two candidate predictor sets were defined before model fitting. The clinical model included age, BMI, menopausal status, histology, grade, FIGO group, tumor size, stromal invasion, LVSI, parametrial invasion, margin status, surgery type, and adjuvant treatment. The immune-extended model added log-transformed SII, stromal TIL percentage, and the integrated immune-inflammatory phenotype to the same clinical variables.

On the training set, we fitted:

(1) a clinical Cox model;

(2) an immune-extended Cox model;

(3) a LASSO-Cox model with 10-fold cross-validation and selection of the penalty parameter by the one-standard-error rule;

(4) a CoxBoost model; and

(5) a random survival forest (RSF) with 1,000 trees and permutation-based variable importance (16–18). Time-dependent discrimination was assessed in the test set at 12, 24, 36, 48, and 60 months using time-dependent ROC analysis (19). Global discrimination was summarized by Harrell’s C-index, and overall prediction error was summarized by the integrated Brier score (IBS). Calibration was examined at prespecified follow-up times using time-specific calibration plots.

As an additional internal validation step, bootstrap resampling was performed on the full cohort to obtain optimism-corrected estimates of model performance. For each bootstrap resample, the median SII cutoff used for phenotype construction was recalculated, and apparent and test performance were compared to estimate optimism.

### 2.8 Decision-analytic evaluation

Potential decision-analytic value was examined using decision curve analysis (DCA) (20). Because the final DCA focused on a fixed prediction horizon, we used a 36-month landmark approach. The landmark cohort included patients who had documented recurrence within 36 months and patients who remained recurrence-free with follow-up beyond 36 months; patients censored before 36 months without recurrence were excluded. Net benefit was then calculated across prespecified threshold probabilities using the predicted 36-month recurrence risk from each model. Key net benefit values at selected thresholds were also tabulated. This landmark design allowed comparison at a fixed time point, but may have introduced selection bias related to differences in follow-up duration.

### 2.9 Explainability analysis

To improve interpretability of the final models, two complementary explainability approaches were used. For the LASSO-Cox model, non-zero coefficients retained at the selected penalty level were extracted, and coefficient-based importance was summarized from the absolute coefficient magnitudes. For the RSF model, variable importance was obtained from the forest-based importance measure. In addition, SHAP (Shapley additive explanations) values were calculated for the predicted 36-month recurrence risk for both the LASSO-Cox model and RSF to quantify the contribution of each feature to individual-level predictions (21). For LASSO-Cox, the linear predictor was converted to 36-month risk through an offset Cox model fitted in the training set. For RSF, SHAP values were calculated directly from the model-predicted 36-month risk.

### 2.10 Sensitivity analyses for redundancy and incremental predictive value

To address potential redundancy between the integrated immune-inflammatory phenotype and its component variables, we performed prespecified sensitivity analyses using four predictor formulations: (1) clinical variables alone, (2) clinical variables plus the integrated immune-inflammatory phenotype alone, (3) clinical variables plus the individual component variables alone (log-transformed SII and stromal TIL percentage), and (4) the combined formulation including both the phenotype and its component variables. These formulations were compared in the test set using Cox, LASSO-Cox, and random survival forest (RSF) models. For the Cox framework, formal incremental value was assessed by likelihood ratio testing and changes in Akaike information criterion (AIC) across nested models. To evaluate the degree of overlap among immune-related predictors in the combined formulation, design-matrix variance inflation factors (VIFs) were calculated. Because nested hypothesis testing is most naturally defined in the Cox setting, the formal incremental comparisons were based primarily on the Cox models, whereas RSF results were used as a complementary nonparametric assessment of redundancy.

### 2.11 Sensitivity analyses for continuous immune-inflammatory representations

To examine whether dichotomizing stromal TILs and SII materially influenced the main findings, we performed prespecified sensitivity analyses using continuous representations of the same immune-inflammatory information. In addition to the categorical integrated immune-inflammatory phenotype, we evaluated a model including log-transformed SII and stromal TIL percentage as continuous predictors, an exploratory continuous immune-inflammatory composite score derived from standardized log-SII and stromal TIL values, and a spline-based Cox model allowing nonlinear effects of both variables. These formulations were compared with the categorical phenotype model using the C-index, 36-month AUC, IBS, likelihood ratio statistics, and AIC.

### 2.12 Sensitivity analyses for independence from disease severity

To further assess whether the association between the poor integrated immune-inflammatory phenotype and recurrence primarily reflected underlying disease severity, we performed additional sensitivity analyses including a FIGO-stratified Cox model, subgroup analyses within broader stage strata (IA–IB and IIA–IIIC), and propensity score overlap-weighted Cox models based on baseline severity-related clinicopathological variables.

### 2.13 Software and statistical threshold

All analyses were performed in R (version 4.4.1). All statistical tests were two-sided, and a *P* value <0.05 was considered statistically significant.

## 3 Results

### 3.1 Baseline characteristics according to recurrence status

A total of 612 patients were included in the final analysis (**Supplementary Figure 1**), of whom 119 experienced an RFS event. Compared with patients without recurrence, those with recurrence had less favorable clinicopathological features, including a more advanced FIGO group, larger tumor size, deeper stromal invasion, more frequent LVSI, a higher rate of lymph node positivity, and more frequent positive surgical margins (**Table 1**). Adjuvant treatment patterns also differed between the two groups, with concurrent chemoradiotherapy being more common among patients with recurrence.

**Table 1:** Baseline clinicopathological characteristics according to recurrence-free survival event status.

Variable	No N = 493	Yes N = 119	*P*-value
Age, years	50.5 (44.1, 57.4)	52.5 (46.1, 58.4)	0.259
BMI, kg/m^2^	23.1 (21.1, 25.4)	23.8 (21.8, 26.0)	0.056
Menopausal status			0.605
Premenopausal	209 (42%)	47 (39%)	
Postmenopausal	284 (58%)	72 (61%)	
Histology			0.643
Squamous cell carcinoma	397 (81%)	98 (82%)	
Adenocarcinoma	71 (14%)	13 (11%)	
Adenosquamous carcinoma	18 (3.7%)	5 (4.2%)	
Other	7 (1.4%)	3 (2.5%)	
Grade			0.668
G1	78 (16%)	19 (16%)	
G2	277 (56%)	62 (52%)	
G3	138 (28%)	38 (32%)	
FIGO group			<0.001
IA	68 (14%)	6 (5.0%)	
IB	318 (65%)	41 (34%)	
IIA	51 (10%)	8 (6.7%)	
IIIC	56 (11%)	64 (54%)	
Tumor size, cm	2.9 (2.2, 3.7)	3.8 (2.8, 4.8)	<0.001
Stromal invasion			0.002
Inner third	111 (23%)	16 (13%)	
Middle third	186 (38%)	35 (29%)	
Outer third/full thickness	196 (40%)	68 (57%)	
LVSI	164 (33%)	65 (55%)	<0.001
Parametrial invasion	26 (5.3%)	9 (7.6%)	0.377
Lymph node status			<0.001
Negative	437 (89%)	55 (46%)	
Positive	56 (11%)	64 (54%)	
Margin status			<0.001
Negative	488 (99%)	111 (93%)	
Positive	5 (1.0%)	8 (6.7%)	
Surgery type			0.571
Open radical hysterectomy	357 (72%)	83 (70%)	
Minimally invasive radical hysterectomy	136 (28%)	36 (30%)	
Adjuvant therapy			<0.001
None	213 (43%)	23 (19%)	
Radiotherapy	188 (38%)	34 (29%)	
Chemotherapy	16 (3.2%)	6 (5.0%)	
Concurrent chemoradiotherapy	76 (15%)	56 (47%)	
Systemic immune-inflammation index (SII)	479.2 (330.7, 707.3)	752.8 (529.5, 1,069.2)	<0.001
Stromal TILs, %	19.5 (6.8, 30.1)	12.9 (1.9, 27.5)	0.017
Integrated immune-inflammatory phenotype			<0.001
Favorable	135 (27%)	12 (10%)	
Intermediate	243 (49%)	44 (37%)	
Poor	115 (23%)	63 (53%)	

Data are presented as median (Q1, Q3) or n (%).

RFS event status was defined according to recurrence-free survival follow-up.

P values were calculated using the Wilcoxon rank-sum test for continuous variables and the Pearson chi-squared test or Fisher’s exact test for categorical variables, as appropriate.

BMI, body mass index; FIGO, International Federation of Gynecology and Obstetrics; LVSI, lymphovascular space invasion; SII, systemic immune-inflammation index; TILs, tumor-infiltrating lymphocytes.

Immune-inflammatory variables showed the same overall direction. Patients with recurrence had higher SII values, lower stromal TIL levels, and a less favorable distribution of the integrated immune-inflammatory phenotype. By contrast, age, menopausal status, histology, grade, parametrial invasion, and surgery type were not significantly different between the two groups (**Table 1**).

### 3.2 Immune-inflammatory stratification of recurrence-free survival

**Figure 1:**
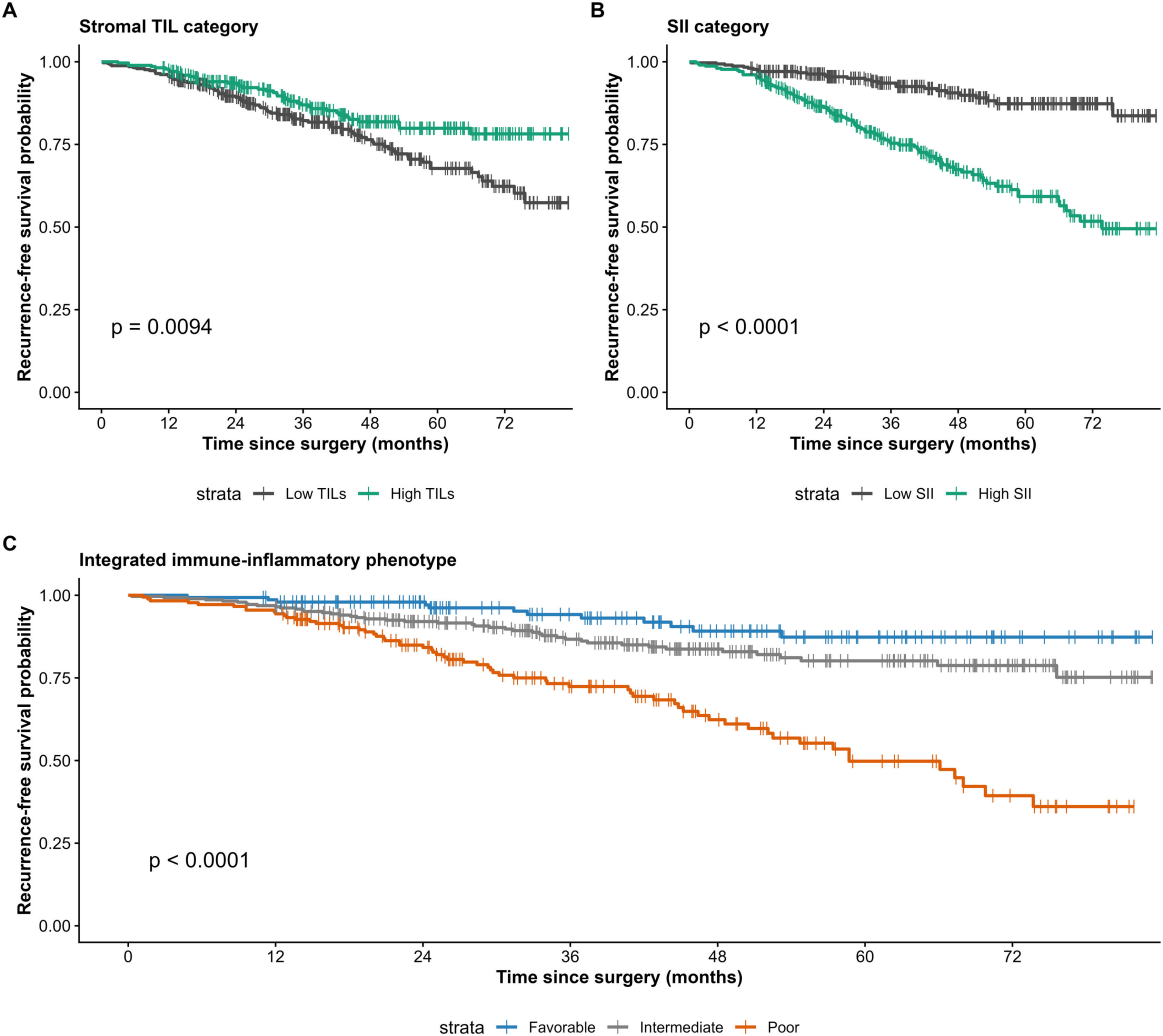
Kaplan-Meier curves for recurrence-free survival according to immune-inflammatory stratification. Kaplan-Meier curves for recurrence-free survival according to **(A)** stromal TIL category, **(B)** SII category, and **(C)** integrated immune-inflammatory phenotype. *P* values were calculated using the log-rank test.

Kaplan-Meier analysis showed that stromal TIL category, SII category, and the integrated immune-inflammatory phenotype all stratified RFS (**Figure 1**). Patients with high stromal TILs had better RFS than those with low TILs (**Figure 1A**). In contrast, patients with high SII had worse RFS than those with low SII (**Figure 1B**). The clearest separation was observed for the integrated immune-inflammatory phenotype, with the favorable group showing the best RFS, the poor group the worst RFS, and the intermediate group lying between them (**Figure 1C**).

Clinicopathological patterns across phenotype groups were consistent with these survival differences. The poor phenotype group was enriched for more advanced FIGO stage, larger tumors, higher lymph node positivity, higher SII, and lower stromal TILs, whereas the favorable phenotype group showed the opposite pattern (**Supplementary Table 1**).

### 3.3 Dose-response association between SII and recurrence risk

Restricted cubic spline analysis demonstrated a significant overall association between SII and recurrence risk (overall *P* < 0.001), whereas the test for nonlinearity was not significant (nonlinear *P* = 0.305; **Supplementary Figure 2**). The hazard of recurrence increased progressively with higher SII values, supporting a broadly monotonic association rather than a marked threshold or U-shaped relationship.

### 3.4 Multivariable predictors of recurrence-free survival

In the multivariable Cox model, FIGO IIIC disease remained strongly associated with worse RFS compared with FIGO IA (HR 7.83, 95% CI 2.90–21.12, *P* < 0.001; **Table 2**). Positive margin status was also independently associated with recurrence (HR 4.15, 95% CI 1.84–9.36, *P* < 0.001). Among the immune-inflammatory variables, the poor integrated immune-inflammatory phenotype remained an independent predictor of worse RFS compared with the favorable phenotype (HR 3.29, 95% CI 1.73–6.26, *P* < 0.001), whereas the intermediate phenotype was not significantly different from the favorable group.

**Table 2:** Multivariable Cox regression analysis for recurrence-free survival.

Variable	HR	95% CI	*P* value
Age, years	0.99	0.97, 1.01	0.435
BMI, kg/m²	1.04	0.98, 1.10	0.177
Histology			
*Squamous cell carcinoma*	—	—	
*Adenocarcinoma*	0.78	0.43, 1.41	0.417
*Adenosquamous carcinoma*	1.72	0.68, 4.35	0.253
*Other*	0.56	0.15, 2.07	0.384
Grade			
*G1*	—	—	
*G2*	1.26	0.74, 2.16	0.392
*G3*	1.26	0.71, 2.22	0.433
FIGO group			
*IA*	—	—	
*IB*	1.50	0.62, 3.61	0.365
*IIA*	1.83	0.62, 5.34	0.272
*IIIC*	7.83	2.90, 21.12	<0.001
LVSI			
*No*	—	—	
*Yes*	1.47	1.00, 2.18	0.052
Margin status			
*Negative*	—	—	
*Positive*	4.15	1.84, 9.36	<0.001
Adjuvant therapy			
*None*	—	—	
*Radiotherapy*	1.36	0.78, 2.39	0.282
*Chemotherapy*	1.96	0.69, 5.55	0.208
*Concurrent chemoradiotherapy*	1.07	0.52, 2.22	0.850
Integrated immune-inflammatory phenotype			
*Favorable*	—	—	
*Intermediate*	1.59	0.83, 3.04	0.161
*Poor*	3.29	1.73, 6.26	<0.001

Hazard ratios (HRs) and 95% confidence intervals (CIs) were estimated using multivariable Cox proportional hazards regression.

Reference categories were squamous cell carcinoma for histology, G1 for grade, FIGO IA for FIGO group, no LVSI, negative margin status, no adjuvant therapy, and favorable integrated immune-inflammatory phenotype.

HR, hazard ratio; CI, confidence interval; FIGO, International Federation of Gynecology and Obstetrics; LVSI, lymphovascular space invasion.

LVSI showed a borderline association with recurrence risk (HR 1.47, 95% CI 1.00–2.18, *P* = 0.052). Age, BMI, histology, grade, and adjuvant therapy category were not independently associated with RFS after adjustment (**Table 2**). To further examine whether the prognostic association of the poor phenotype was driven mainly by disease severity, we performed stage-stratified and propensity score overlap-weighted analyses (**Supplementary Table 7**). The association between poor phenotype and worse recurrence-free survival remained significant in the FIGO-stratified model, was directionally consistent across broader stage strata, and persisted after overlap weighting.

### 3.5 Performance comparison of survival models

**Figure 2:**
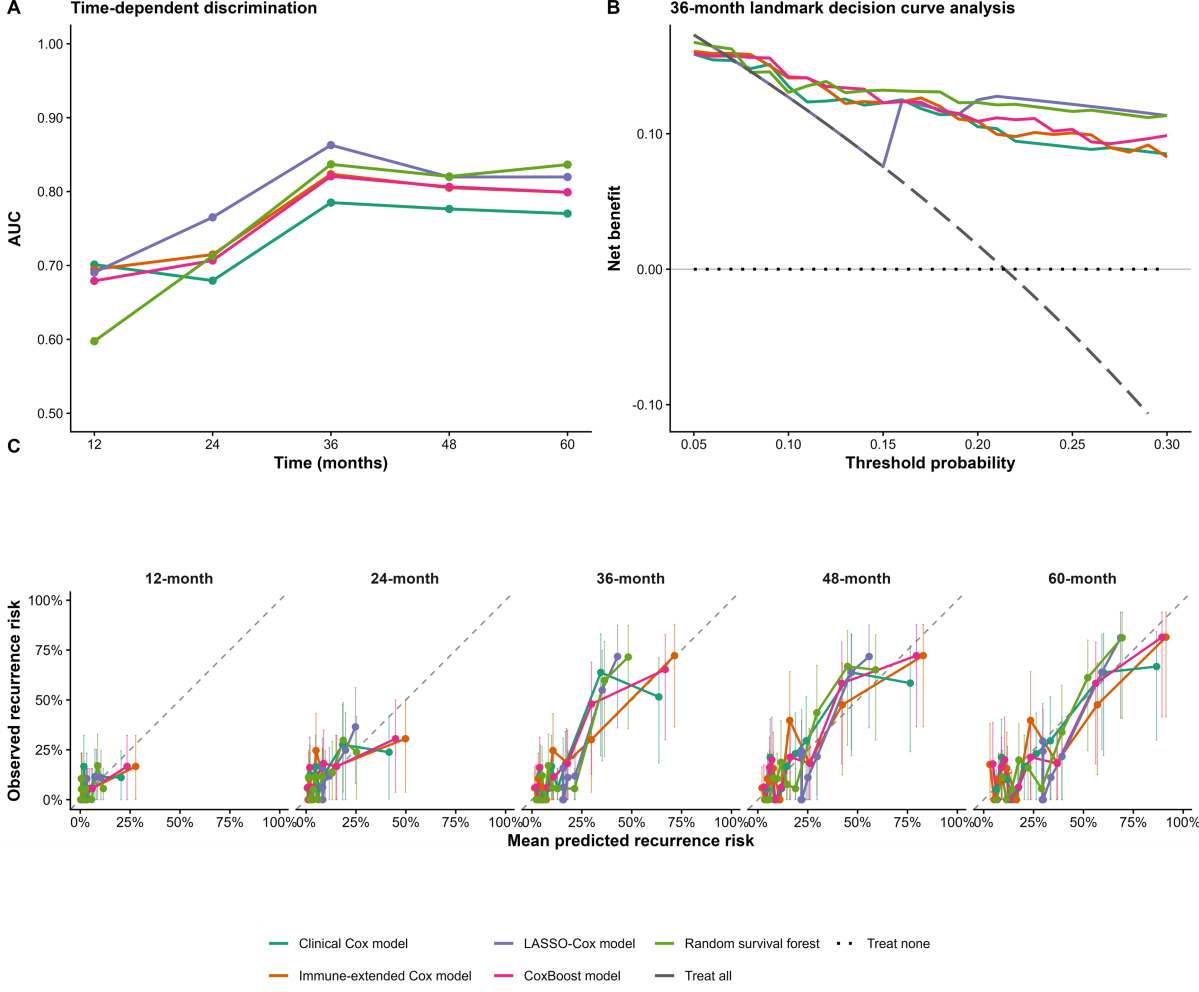
Comparison of prognostic performance across survival models. **(A)** Time-dependent AUCs at 12, 24, 36, 48, and 60 months. **(B)** Decision curve analysis at the 36-month landmark horizon. **(C)** Time-specific calibration plots. LASSO-Cox showed the strongest mid-term discrimination, whereas random survival forest performed best at longer follow-up. Decision curve analysis suggested potential net benefit for model-based postoperative risk stratification across selected threshold probabilities.

We next compared the prognostic performance of several survival models in the test set (**Table 3**; **Figures 2A, C**). The clinical Cox model yielded a C-index of 0.738. Extending the model with immune-inflammatory variables increased the C-index to 0.756 and resulted in the lowest IBS (0.278), indicating the lowest overall prediction error among the evaluated models (**Table 3**).

**Table 3:** Comparison of prognostic performance across survival models.

**Model**	**C-index**	**12-month AUC**	**24-month AUC**	**36-month AUC**	**48-month AUC**	**60-month AUC**	**IBS**
Clinical Cox model	0.738	**0.701**	0.680	0.785	0.776	0.770	0.310
Clinical + immune phenotype Cox model	0.756	0.695	0.715	0.824	0.805	0.799	**0.278**
LASSO-Cox model	**0.782**	0.691	**0.765**	**0.863**	**0.820**	0.820	0.302
CoxBoost model	0.752	0.679	0.707	0.821	0.806	0.799	0.293
Random survival forest	0.779	0.598	0.713	0.837	0.820	**0.837**	0.324

Model performance was evaluated in the test set.

Higher C-index and time-dependent AUC values indicate better discrimination, whereas a lower integrated Brier score (IBS) indicates better overall prediction accuracy.

IBS was calculated by integrating inverse probability of censoring weighted Brier scores across the prespecified evaluation times of 12, 24, 36, 48, and 60 months.

AUC, area under the curve; IBS, integrated Brier score; RSF, random survival forest. Bold values indicate the optimal performance among all compared models for each evaluation metric.

Among the more flexible models, LASSO-Cox showed the highest C-index (0.782) and achieved the best discrimination at 24 and 36 months, with AUCs of 0.765 and 0.863, respectively. RSF showed the highest 60-month AUC (0.837) and maintained competitive performance at later time points. CoxBoost showed intermediate performance across most metrics. Overall, no single model was uniformly superior across all indices, but regularized and machine learning-based models generally improved discrimination beyond the conventional clinical Cox model, while the immune-extended Cox model yielded the lowest overall prediction error (**Table 3**; **Figure 2A**). Time-specific calibration plots showed generally acceptable agreement between predicted and observed recurrence risk across prespecified time points, although greater variability was seen at lower predicted risk ranges and at earlier time points, where event numbers were limited (**Figure 2C**). In additions, bootstrap internal validation using 1,000 resamples showed attenuation of model discrimination after optimism correction, but the relative ranking of the candidate models was broadly preserved, with RSF retaining the highest optimism-corrected C-index and 36-month AUC (**Supplementary Table 4**).

In sensitivity analyses for predictor redundancy, both the phenotype-only and components-only formulations improved performance over the clinical model, and the combined formulation retained statistically significant incremental value over either formulation alone; however, the absolute gain in discrimination was modest in the Cox framework. In RSF, both the phenotype-only and components-only formulations also outperformed the clinical formulation, whereas the combined formulation yielded only small further improvements. Collinearity diagnostics in the combined Cox formulation showed expected overlap among the immune-related terms, with moderate variance inflation for the poor phenotype indicator and acceptable values for the remaining immune-related variables, supporting partial but not complete redundancy between the composite phenotype and its component variables.

To assess whether dichotomization materially influenced the main findings, we compared the categorical phenotype with continuous formulations of the same immune-inflammatory information (**Supplementary Table 5**). All immune-inflammatory formulations improved model fit relative to the clinical model. The categorical phenotype showed the strongest discrimination in the test set, with the highest C-index (0.732) and 36-month AUC (0.794), whereas the continuous component-based and spline-based formulations yielded the lowest IBS (both 0.134). The spline-based model showed the largest likelihood-based improvement over the clinical model, but this did not translate into superior test-set discrimination compared with the categorical phenotype. Overall, these analyses support the robustness of the main findings while indicating that the categorical phenotype should be interpreted as a pragmatic summary of an underlying continuous immune-inflammatory gradient rather than as a strict biologic threshold-based construct.

### 3.6 Decision-analytic evaluation

Decision-analytic performance was assessed using a 36-month landmark DCA (**Figure 2B**; **Supplementary Table 2**). Across clinically relevant threshold probabilities, all model-based strategies retained positive net benefit, although the magnitude of benefit varied by threshold. At lower thresholds, differences between models were modest. At intermediate-to-higher thresholds, particularly between 20% and 30%, the LASSO-Cox model and RSF showed the most favorable and stable net benefit profiles. The treat-all strategy declined rapidly with increasing threshold and became unfavorable at higher thresholds. The immune-extended Cox model showed modest improvement over the clinical Cox model across part of the threshold range, but no model was uniformly superior at all thresholds (**Figure 2B**; **Supplementary Table 2**).

### 3.7 Explainability of the LASSO-Cox and RSF models

**Figure 3:**
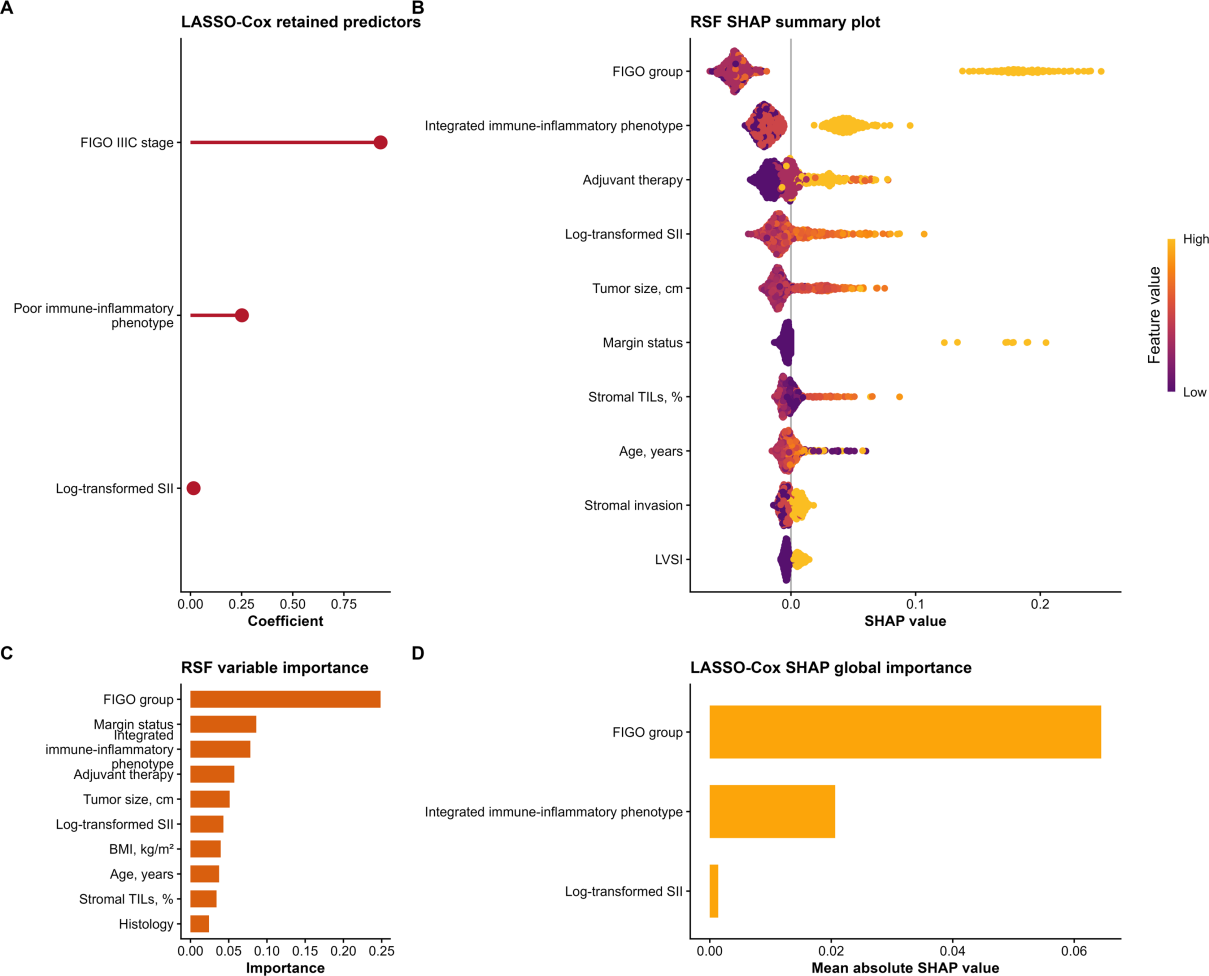
Explainability of the LASSO-Cox and random survival forest models. **(A)** Predictors retained in the LASSO-Cox model. **(B)** SHAP summary plot for the random survival forest model. **(C)** Variable importance ranking for the random survival forest model. **(D)** SHAP-based global importance for the LASSO-Cox model. These analyses indicate that disease extent and immune-inflammatory features were the main contributors to recurrence risk prediction.

Explainability analyses highlighted both shared and model-specific patterns (**Figure 3**). The LASSO-Cox model retained only three predictors: FIGO IIIC stage, poor integrated immune-inflammatory phenotype, and log-transformed SII (**Figures 3A, D**). Among these, FIGO IIIC stage contributed most strongly, followed by poor phenotype, whereas the contribution of log-transformed SII was smaller but retained after penalized selection.

By contrast, the RSF model captured a broader prognostic structure. Variable importance ranking placed FIGO group first, followed by margin status, integrated immune-inflammatory phenotype, adjuvant therapy, tumor size, and log-transformed SII (**Figure 3C**). SHAP-based interpretation further supported a major contribution of disease extent and immune-inflammatory features to 36-month recurrence risk at the individual level (**Figure 3B**). Taken together, these findings suggest that the LASSO-Cox model provided a compact and clinically interpretable risk representation, whereas RSF captured a more complex risk structure involving multiple clinicopathological and immune-inflammatory variables.

### 3.8 Tissue-level immunohistochemical validation

**Figure 4:**
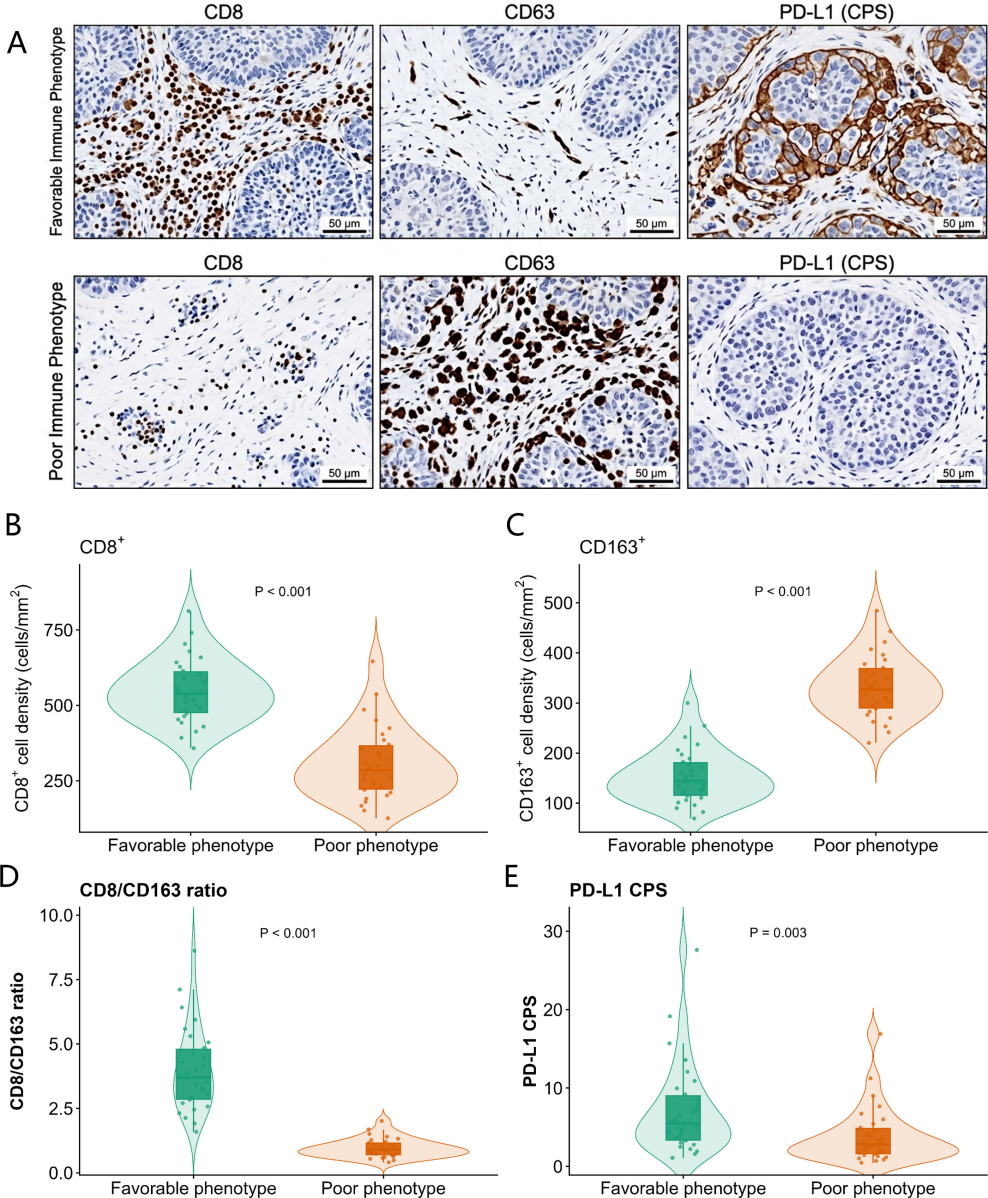
Immunohistochemical validation of tissue immune markers according to integrated immune-inflammatory phenotype. **(A)** Illustrative representative immune staining patterns in the favorable and poor phenotype groups. **(B)** CD8+ cell density. **(C)** CD163+ cell density. **(D)** CD8/CD163 ratio. **(E)** PD-L1 combined positive score (CPS). Quantitative comparisons were performed between the favorable and poor phenotype groups.

To examine whether the integrated immune-inflammatory phenotype corresponded to distinct tissue immune states, we compared immunohistochemical markers between the favorable and poor phenotype groups (**Table 4**, **Figure 4**, **Supplementary Figure 2**). The favorable phenotype group showed markedly higher CD8+ cell density and lower CD163+ cell density than the poor phenotype group. Consistently, the CD8/CD163 ratio was substantially higher in the favorable group. PD-L1 CPS was also higher in the favorable phenotype group (**Table 4**, **Figure 4**). This finding was interpreted in the context of the broader immune profile of that group, rather than as indicating an intrinsically favorable prognostic role for PD-L1 itself.

**Table 4:** Immunohistochemical validation of tissue immune markers according to integrated immune-inflammatory phenotype.

Marker	Favorable N = 30	Poor N = 30	*P*-value
CD8+ cell density, cells/mm^2^	539.5 (499.6, 647.7)	285.4 (202.3, 339.6)	<0.001
CD163+ cell density, cells/mm^2^	144.5 (108.4, 172.3)	326.9 (283.3, 364.3)	<0.001
CD8/CD163 ratio	3.7 (3.1, 5.3)	0.9 (0.6, 1.0)	<0.001
PD-L1 CPS	5.5 (3.2, 8.9)	2.8 (1.6, 5.0)	0.003

Data are presented as median (Q1, Q3).

Comparisons were performed between the favorable and poor integrated immune-inflammatory phenotype groups using the Wilcoxon rank-sum test.

CPS, combined positive score; PD-L1, programmed death-ligand 1.

In the immunohistochemical validation subset, higher log-transformed SII was strongly associated with lower CD8+ cell density, higher CD163+ cell density, and a lower CD8/CD163 ratio (all *P* < 0.001; **Supplementary Table 6**, **Supplementary Figure 3**). These associations remained significant after adjustment for FIGO group and tumor size. By contrast, the inverse association between log-SII and PD-L1 CPS was weaker in the unadjusted analysis and was not retained after adjustment.

## 4 Discussion

The present study supports a simple but clinically meaningful point: recurrence after primary surgery for cervical cancer is not determined by anatomical extent alone. In our cohort, recurrence clustered in patients with more advanced FIGO group, larger tumors, deeper stromal invasion, LVSI, nodal involvement, positive margins, higher SII, lower stromal TILs, and a less favorable integrated immune-inflammatory phenotype. After adjustment, FIGO IIIC disease, positive margin status, and the poor integrated immune-inflammatory phenotype remained the strongest correlates of worse RFS. The consistency of these findings across conventional survival analysis, multivariable modeling, model comparison, decision-analytic evaluation, and tissue validation suggests that the integrated phenotype is capturing a biologically meaningful component of recurrence risk rather than a purely statistical construct.

This framework is clinically plausible. Even in early-stage disease, recurrent cervical cancer remains a substantial problem after primary treatment, and efforts to refine postoperative risk stratification remain active because conventional clinicopathological variables do not fully explain the heterogeneity of outcome (22). The dominant role of stage in our data is expected and remains aligned with validation studies of the 2018 FIGO staging system, in which stage separation retained strong prognostic relevance, while outcomes within stage IIIC remained heterogeneous and partly dependent on local tumor factors (23). Recent pathology-focused reviews have likewise emphasized that tumor size, depth of stromal invasion, LVSI, nodal status, and tumor-free distance still define the backbone of cervical cancer prognostication after surgery (4). Margin status fits the same pattern. Prior surgical series have shown that close or positive margins track with other high-risk features and are linked to worse outcome, even when they are difficult to isolate completely from the rest of the pathological risk profile (24, 25). Our multivariable results are therefore clinically coherent: the integrated immune-inflammatory phenotype added information, but it did so on top of, not instead of, strong conventional disease burden markers.

The SII findings also fit well with the current literature. Elevated pretreatment SII has repeatedly been associated with worse outcomes in cervical cancer, including in radiotherapy-treated cohorts (9). Two recent meta-analyses reached the same overall conclusion and support SII as a reproducible prognostic signal across different treatment settings and study designs (10, 26). What is useful in our data is that the spline analysis did not suggest a clear nonlinear threshold effect. The association was significant overall, but the nonlinearity test was not. That makes the result easier to interpret clinically. Rather than implying a narrow cut-point with special biological meaning, our findings support the view that SII behaves as a graded marker of inflammatory burden, with progressively higher recurrence risk as systemic inflammation rises. That interpretation is also consistent with the wider literature on hemato-immunological markers in cervical cancer, where inflammation-related indices tend to function as continuous burden markers rather than sharply dichotomous biological states (9, 26, 27).

The TIL results reinforce the local side of that same biology. Stromal TILs have been increasingly recognized as more informative than intraepithelial lymphocytes in cervical cancer, which is relevant because our analysis focused on stromal TIL assessment rather than a broader, mixed immune count (10). Other cervical cancer studies have likewise shown that abundant lymphocytic infiltration, especially CD8-positive populations, is associated with better survival or treatment response in patients receiving chemoradiotherapy or definitive radiotherapy (8, 28). Earlier work also showed that cervical tumors enriched with CD8-positive T cells and specific myeloid populations were associated with more favorable survival (29). Our data are very much in line with that pattern. High TILs alone separated RFS, but the integrated phenotype separated it more clearly. That is an important distinction. Local immune infiltration and systemic inflammatory activation do not measure the same process. One reflects the state of the tumor microenvironment; the other reflects the host systemic response. Combining them appears to better capture the balance between antitumor immunity and protumor inflammation than either marker alone.

The tissue validation data make that interpretation more convincing. Favorable phenotype tumors showed higher CD8-positive cell density, lower CD163-positive cell density, and a substantially higher CD8/CD163 ratio than poor phenotype tumors. This is exactly the pattern one would expect if the favorable phenotype corresponded to a more immune-active microenvironment and the poor phenotype to a more suppressive one. The macrophage findings are especially relevant. In cervical cancer, CD163-positive M2-like macrophage infiltration has been linked to PD-L1 expression and to more aggressive biological behavior (28, 30). Dynamic analyses during treatment have also associated higher CD163 and PD-L1 expression in the tumor microenvironment with poorer short-term progression outcomes (31). More broadly, work on the immunologic effects of chemoradiation in cervical cancer has highlighted that macrophage composition, T-cell infiltration, and checkpoint expression are interconnected rather than independent features (5). Our data fit naturally into that framework: the poor integrated phenotype was not only statistically adverse, it corresponded to a tissue immune contexture that appeared more suppressive and less cytotoxic.

The PD-L1 result is more nuanced, but still biologically credible. In our cohort, PD-L1 CPS was higher in the favorable phenotype group. This should not be read too simplistically. In cervical cancer, PD-L1 does not function as a universal marker of immune failure. Its meaning depends heavily on whether expression is occurring in tumor cells, immune cells, or both, and whether it sits within an inflamed, T-cell-rich environment or a macrophage-dominant suppressive niche (32, 33). Several cervical cancer studies have shown that PD-L1 expression can coexist with higher CD8 infiltration and, in some settings, identify a subgroup with better or at least distinct prognosis rather than uniformly worse outcome (34, 35). Our result is compatible with that view. In the favorable phenotype, higher CPS appeared alongside higher CD8 density and a higher CD8/CD163 ratio, which is more consistent with an immune-engaged or “inflamed” phenotype than with immune exclusion. At the same time, the broader clinical relevance of PD-L1 in cervical cancer is now established by pembrolizumab-based therapy in recurrent or metastatic disease (36, 37). The implication here is not that higher PD-L1 is intrinsically favorable, but that PD-L1 should be interpreted in the context of the surrounding immune architecture rather than as a single-direction biomarker.

The model comparison results also deserve a measured interpretation. We did not identify a single model that dominated all performance metrics. The immune-extended Cox model had the lowest IBS, suggesting the best overall average prediction error. LASSO-Cox achieved the highest C-index and the best 24- and 36-month discrimination, whereas RSF performed best at 60 months. This pattern is not contradictory. It reflects the fact that different models emphasize different properties of the data. The LASSO-Cox model is designed to shrink coefficients and remove weaker variables, which often improves stability and interpretability when the signal is concentrated in a small number of features (17). RSF, by contrast, is better able to accommodate nonlinear effects, variable interactions, and layered risk structures that are not easily captured by a sparse linear predictor (18). That contrast was visible in our explainability analysis. LASSO-Cox retained only three predictors—FIGO IIIC stage, poor integrated immune-inflammatory phenotype, and log-transformed SII—whereas RSF distributed importance more broadly across stage, margin status, integrated phenotype, adjuvant treatment, tumor size, and immune-inflammatory variables. In this sense, the two models were less rivals than complements: LASSO-Cox provided a compact and clinically readable signal, whereas RSF preserved a wider view of prognostic complexity. LASSO-Cox and RSF may have complementary practical value: LASSO-Cox offers a more parsimonious and interpretable risk model, whereas RSF provides greater flexibility for capturing complex prognostic structure. This interpretation is also consistent with prior cervical cancer work showing that machine-learning survival models can outperform conventional Cox models in selected settings, while still raising the usual need for careful validation and transparent interpretation (38). Because the integrated immune-inflammatory phenotype was constructed from stromal TILs and SII, some degree of overlap with its component variables was expected. In sensitivity analyses, both the phenotype alone and the component variables alone were prognostically informative, whereas the combined formulation yielded statistically significant but quantitatively modest additional gain and showed moderate collinearity among immune-related terms. Accordingly, the combined models should be interpreted as exploratory representations of overlapping immune-inflammatory information rather than as evidence that each term contributes fully independent biological signal.

The decision curve analysis adds a practical perspective to the model comparison, but it should be interpreted cautiously. DCA estimates theoretical net benefit under a given set of threshold probabilities and does not show that using the model in practice would improve patient outcomes. In our 36-month landmark analysis, differences between models were small at low thresholds, whereas LASSO-Cox and RSF showed more stable net benefit at intermediate thresholds, particularly around 20% to 30%. This may be the most clinically relevant range, as it is closer to the situation in which the question is not whether all patients should undergo close follow-up, but which patients may warrant intensified surveillance, earlier imaging reassessment, or more individualized postoperative management. At the same time, the landmark design excluded patients censored before 36 months without recurrence, which may have introduced selection bias and influenced the apparent net benefit estimates. For these reasons, the DCA findings are better viewed as indicating potential decision-analytic value rather than established clinical utility.

From a translational perspective, the integrated immune-inflammatory phenotype may be most useful as a pragmatic postoperative risk-stratification tool rather than as a stand-alone determinant of adjuvant therapy. In clinical practice, such a phenotype could help identify patients who may benefit from closer surveillance intensity, more individualized follow-up planning, or more careful multidisciplinary discussion when standard clinicopathological risk factors do not fully capture recurrence concern. At the same time, our findings should not be interpreted as sufficient to support phenotype-guided adjuvant treatment decisions on their own. Any such application would require external validation and prospective evaluation.

Several limitations should be acknowledged. First, this was a single-center retrospective cohort, so the findings require external validation before broader use. Although no independent external cohort was available, we supplemented the train-test split analysis with bootstrap internal validation and optimism-corrected performance estimates. Nevertheless, this cannot substitute for true external validation, particularly given the cohort-derived thresholds used for SII and phenotype construction. Second, the integrated phenotype was deliberately pragmatic, built from accessible markers rather than high-dimensional molecular profiling. That makes it easier to apply, but also means that threshold definitions may remain cohort-sensitive. Stromal TILs and SII were dichotomized for construction of the integrated phenotype. Although this improved interpretability and facilitated pragmatic risk stratification, both variables are intrinsically continuous and their biological effects are unlikely to be fully threshold-based. In our sensitivity analyses, continuous and spline-based formulations yielded broadly consistent results and improved model fit, suggesting that the main prognostic signal was not solely an artifact of categorization. At the same time, the categorical phenotype retained the strongest test-set discrimination, supporting its use as a clinically convenient summary of an underlying continuous immune-inflammatory spectrum. Third, the immunohistochemical validation cohort was relatively small. The tissue validation component was based on a focused marker panel and exploratory correlative analyses. Although these analyses showed directional alignment between higher preoperative SII and a more suppressive tissue immune context, they do not establish causality or define the mechanistic pathway linking systemic inflammation to local immune architecture. Larger-scale tissue validation, ideally in independent cohorts, will be needed to confirm the observed immune marker patterns. Fourth, the 36-month landmark decision curve analysis excluded patients censored before 36 months without recurrence, which may have introduced selection bias and affected model evaluation. Fifth, because PD-L1 was evaluated by single-marker immunohistochemistry, we could not distinguish tumor cell-associated from immune cell-associated PD-L1 expression, nor could we determine whether CD163+ macrophages represented the dominant PD-L1-positive population. Finally, the study was based on baseline variables. Dynamic changes in inflammatory indices, immune infiltration, or checkpoint expression during treatment may add further information and deserve future study (31).

Despite these limitations, the study has a clear practical message. In surgically treated cervical cancer, recurrence risk is shaped by both tumor extent and immune-inflammatory context. A simple integrative phenotype combining stromal TILs and SII remained independently associated with RFS after adjustment for standard clinicopathological factors, showed value in survival stratification and decision analysis, and was supported by tissue-level immune differences. That combination of accessibility, prognostic relevance, and biological plausibility is what makes the integrated immune-inflammatory phenotype worth carrying forward into external validation and, potentially, into clinically usable postoperative risk assessment tools.

## Data Availability

The raw data supporting the conclusions of this article will be made available by the authors, without undue reservation.
