# Supplementary material for: Prognostic value of an integrated immune-inflammatory phenotype in surgically treated cervical cancer: survival modeling and immunohistochemical validation
Source: Front Immunol. 2026 Jun 16;17:1850891. doi: 10.3389/fimmu.2026.1850891 (PMC13314612; doi:10.3389/fimmu.2026.1850891)
Supplement: Supplementary file 1 [file Table1.docx]

**Supplementary Table S1. Clinicopathological characteristics across integrated immune-inflammatory phenotype groups.**

| **Variable** | **Favorable**  N = 147^1^ | **Intermediate**  N = 287 | **Poor**  N = 178 | ***P*-value** |
| --- | --- | --- | --- | --- |
| **Age, years** | 50.3 (45.0, 57.4) | 50.5 (44.0, 57.2) | 51.8 (44.2, 58.6) | 0.8 |
| **BMI, kg/m^2^** | 22.9 (21.0, 25.8) | 23.3 (21.2, 25.4) | 23.4 (21.4, 25.8) | 0.7 |
| **Menopausal status** |  |  |  | 0.4 |
| Premenopausal | 56 (38%) | 128 (45%) | 72 (40%) |  |
| Postmenopausal | 91 (62%) | 159 (55%) | 106 (60%) |  |
| **Histology** |  |  |  | 0.6 |
| Squamous cell carcinoma | 119 (81%) | 237 (83%) | 139 (78%) |  |
| Adenocarcinoma | 19 (13%) | 37 (13%) | 28 (16%) |  |
| Adenosquamous carcinoma | 6 (4.1%) | 11 (3.8%) | 6 (3.4%) |  |
| Other | 3 (2.0%) | 2 (0.7%) | 5 (2.8%) |  |
| **Grade** |  |  |  | 0.7 |
| G1 | 25 (17%) | 40 (14%) | 32 (18%) |  |
| G2 | 83 (56%) | 159 (55%) | 97 (54%) |  |
| G3 | 39 (27%) | 88 (31%) | 49 (28%) |  |
| **FIGO group** |  |  |  | <0.001 |
| IA | 18 (12%) | 37 (13%) | 19 (11%) |  |
| IB | 102 (69%) | 175 (61%) | 82 (46%) |  |
| IIA | 15 (10%) | 26 (9.1%) | 18 (10%) |  |
| IIIC | 12 (8.2%) | 49 (17%) | 59 (33%) |  |
| **Tumor size, cm** | 2.9 (2.2, 3.7) | 2.9 (2.2, 3.9) | 3.4 (2.5, 4.3) | 0.003 |
| **Stromal invasion** |  |  |  | 0.2 |
| Inner third | 26 (18%) | 72 (25%) | 29 (16%) |  |
| Middle third | 57 (39%) | 97 (34%) | 67 (38%) |  |
| Outer third/full thickness | 64 (44%) | 118 (41%) | 82 (46%) |  |
| **LVSI** | 53 (36%) | 96 (33%) | 80 (45%) | 0.043 |
| **Parametrial invasion** | 7 (4.8%) | 15 (5.2%) | 13 (7.3%) | 0.6 |
| **Lymph node status** |  |  |  | <0.001 |
| Negative | 135 (92%) | 238 (83%) | 119 (67%) |  |
| Positive | 12 (8.2%) | 49 (17%) | 59 (33%) |  |
| **Margin status** |  |  |  | 0.2 |
| Negative | 145 (99%) | 283 (99%) | 171 (96%) |  |
| Positive | 2 (1.4%) | 4 (1.4%) | 7 (3.9%) |  |
| **Surgery type** |  |  |  | 0.7 |
| Open radical hysterectomy | 104 (71%) | 204 (71%) | 132 (74%) |  |
| Minimally invasive radical hysterectomy | 43 (29%) | 83 (29%) | 46 (26%) |  |
| **Adjuvant therapy** |  |  |  | 0.007 |
| None | 54 (37%) | 121 (42%) | 61 (34%) |  |
| Radiotherapy | 61 (41%) | 105 (37%) | 56 (31%) |  |
| Chemotherapy | 8 (5.4%) | 9 (3.1%) | 5 (2.8%) |  |
| Concurrent chemoradiotherapy | 24 (16%) | 52 (18%) | 56 (31%) |  |
| **Systemic immune-inflammation index (SII)** | 341.0 (244.9, 424.9) | 492.2 (346.7, 734.8) | 791.5 (645.6, 1,058.7) | <0.001 |
| **Stromal TILs, %** | 30.4 (25.3, 37.3) | 18.1 (6.7, 30.1) | 5.9 (1.0, 12.7) | <0.001 |

Data are presented as median (Q1, Q3) or n (%).

P values were calculated using the Kruskal-Wallis rank-sum test for continuous variables and the Pearson chi-squared test or Fisher’s exact test for categorical variables, as appropriate.

**Abbreviations:** BMI, body mass index; FIGO, International Federation of Gynecology and Obstetrics; LVSI, lymphovascular space invasion; SII, systemic immune-inflammation index; TILs, tumor-infiltrating lymphocytes.

**Supplementary Table S2. Net benefit at key thresholds in the 36-month landmark decision curve analysis.**

| **Model** | **Net benefit at 10%** | **Net benefit at 20%** | **Net benefit at 25%** | **Net benefit at 30%** |
| --- | --- | --- | --- | --- |
| Clinical Cox model | 0.135 | 0.105 | 0.090 | 0.085 |
| Immune-extended Cox model | 0.141 | 0.109 | 0.101 | 0.083 |
| LASSO-Cox model | 0.127 | 0.125 | 0.122 | 0.113 |
| CoxBoost model | 0.142 | 0.109 | 0.103 | 0.099 |
| Random survival forest | 0.131 | 0.123 | 0.116 | 0.113 |
| Treat none | 0.000 | 0.000 | 0.000 | 0.000 |
| Treat all | 0.127 | 0.018 | -0.048 | -0.122 |

Net benefit values were calculated in the 36-month landmark cohort.

Patients who experienced recurrence within 36 months and those followed beyond 36 months without recurrence were included. Patients censored before 36 months without recurrence were excluded.

Higher net benefit indicates greater potential clinical utility at the corresponding threshold probability.

**Supplementary Table S3. Redundancy sensitivity analyses for the integrated immune-inflammatory phenotype and its component variables.**

**A. Test-set performance across four predictor formulations**

| **Algorithm** | **Formulation** | **C-index** | **36-month AUC** | **IBS** | **Δ C-index vs clinical** | **Δ 36-month AUC vs clinical** |
| --- | --- | --- | --- | --- | --- | --- |
| Cox | Clinical only | 0.738 | 0.785 | 0.310 | 0.000 | 0.000 |
|  | Phenotype alone | 0.751 | 0.808 | 0.285 | 0.013 | 0.023 |
|  | Components alone | 0.772 | 0.825 | 0.315 | 0.034 | 0.039 |
|  | Both combined | 0.756 | 0.824 | 0.278 | 0.018 | 0.038 |
| RSF | Clinical only | 0.753 | 0.796 | 0.311 | 0.000 | 0.000 |
|  | Phenotype alone | 0.777 | 0.824 | 0.314 | 0.024 | 0.028 |
|  | Components alone | 0.772 | 0.823 | 0.323 | 0.019 | 0.027 |
|  | Both combined | 0.779 | 0.837 | 0.324 | 0.025 | 0.040 |

C-index and 36-month AUC indicate discrimination performance in the test set.

Lower integrated Brier score (IBS) indicates better overall prediction accuracy.

LASSO-Cox is not shown in this table because reduced-formulation fitting was unstable in sensitivity analyses and was not used for formal inference.

**B. Incremental value of nested Cox formulations**

| **Comparison** | **LR χ²** | **df** | ***P* value** | **ΔAIC** |
| --- | --- | --- | --- | --- |
| Clinical only vs phenotype alone | 21.544 | 2 | <0.001 | 17.544 |
| Clinical only vs components alone | 16.726 | 2 | <0.001 | 12.726 |
| Clinical only vs both combined | 29.277 | 4 | <0.001 | 21.277 |
| Phenotype alone vs both combined | 7.733 | 2 | 0.021 | 3.733 |
| Components alone vs both combined | 12.550 | 2 | 0.002 | 8.550 |

Likelihood ratio tests and changes in AIC were used to assess incremental value in nested Cox models. Positive ΔAIC indicates lower AIC for the larger model.

**C. Collinearity diagnostics for the combined Cox formulation.**

| **Term** | **R²** | **VIF** |
| --- | --- | --- |
| Poor phenotype indicator | 0.835 | 6.067 |
| Intermediate phenotype indicator | 0.672 | 3.049 |
| Stromal TILs (%) | 0.582 | 2.392 |
| Log-transformed SII | 0.568 | 2.314 |

Variance inflation factors (VIFs) were calculated from the design matrix of the combined formulation. Values in the moderate range indicate expected overlap among related immune-inflammatory predictors.

**Supplementary Table S4. Bootstrap optimism-corrected performance of candidate survival models.**

| **Model** | **Apparent C-index** | **Optimism-corrected C-index** | **Apparent 36-month AUC** | **Optimism-corrected 36-month AUC** |
| --- | --- | --- | --- | --- |
| Clinical Cox model | 0.776 | 0.735 | 0.826 | 0.786 |
| Immune-extended Cox model | 0.793 | 0.753 | 0.837 | 0.799 |
| LASSO-Cox model | 0.704 | 0.698 | 0.757 | 0.752 |
| CoxBoost model | 0.789 | 0.756 | 0.836 | 0.804 |
| Random survival forest | 0.886 | 0.824 | 0.941 | 0.879 |

Internal validation was performed using 1000 bootstrap resamples on the full cohort.

For each bootstrap resample, apparent performance in the bootstrap sample and test performance in the original cohort were compared to estimate optimism.

Optimism-corrected performance was calculated as apparent performance minus mean optimism.

The SII median cutoff used for phenotype construction was recalculated within each bootstrap resample.

**Supplementary Table S5. Continuous sensitivity analyses of stromal TILs and SII.**

**A. Test-set performance of categorical and continuous immune-inflammatory formulations.**

| **Formulation** | **C-index** | **36-month AUC** | **IBS** | **Δ C-index vs clinical** | **Δ 36-month AUC vs clinical** | **ΔAIC vs clinical** |
| --- | --- | --- | --- | --- | --- | --- |
| Clinical only | 0.697 | 0.765 | 0.154 | 0.000 | 0.000 | 0.000 |
| Categorical phenotype | 0.732 | 0.794 | 0.143 | 0.035 | 0.029 | 9.554 |
| Continuous components | 0.714 | 0.786 | 0.134 | 0.017 | 0.021 | 10.644 |
| Continuous composite score | 0.715 | 0.770 | 0.143 | 0.018 | 0.005 | 4.793 |
| Spline-based continuous model | 0.703 | 0.776 | 0.134 | 0.006 | 0.011 | 12.922 |

Data are presented as performance metrics in the test set.

Higher C-index and 36-month AUC values indicate better discrimination, whereas a lower integrated Brier score (IBS) indicates better overall prediction accuracy.

The continuous composite score was constructed from standardized log-transformed SII and stromal TIL values.

The spline-based continuous model treated log-transformed SII and stromal TIL percentage as continuous nonlinear predictors.

Δ values indicate the change relative to the clinical model.

**B. Incremental value relative to the clinical model.**

| **Comparison** | **LR χ²** | **df** | **P value** | **ΔAIC** |
| --- | --- | --- | --- | --- |
| Clinical vs categorical phenotype | 13.554 | 2 | 0.001 | 9.554 |
| Clinical vs continuous components | 14.644 | 2 | <0.001 | 10.644 |
| Clinical vs continuous composite score | 6.793 | 1 | 0.009 | 4.793 |
| Clinical vs spline-based continuous model | 28.922 | 8 | <0.001 | 12.922 |

Likelihood ratio (LR) tests and changes in Akaike information criterion (ΔAIC) were used to evaluate the incremental value of each immune-inflammatory formulation relative to the clinical model.

Positive ΔAIC values indicate lower AIC, and therefore better fit, for the expanded model compared with the clinical model.

**Abbreviations:** AUC, area under the curve; IBS, integrated Brier score; LR, likelihood ratio; SII, systemic immune-inflammation index; TILs, tumor-infiltrating lymphocytes.

**Supplementary Table 6. Exploratory associations between preoperative SII and tissue immune markers in the immunohistochemical validation subset.**

**A. Spearman correlations.**

| **Marker** | **Spearman** $\rho$ | ***P* value** |
| --- | --- | --- |
| CD8+ cell density | -0.698 | <0.001 |
| CD163+ cell density | 0.722 | <0.001 |
| CD8/CD163 ratio | -0.721 | <0.001 |
| PD-L1 CPS | -0.274 | 0.034 |

Spearman correlations between preoperative log-transformed SII and tissue immune markers in the immunohistochemical validation subset.

**B. Exploratory adjusted linear models.**

| **Outcome** | **β for log-SII** | **95% CI lower** | **95% CI upper** | ***P* value** |
| --- | --- | --- | --- | --- |
| CD8+ cell density | -152.708 | -213.861 | -91.556 | <0.001 |
| CD163+ cell density | 99.034 | 66.264 | 131.805 | <0.001 |
| CD8/CD163 ratio | -2.259 | -3.152 | -1.367 | <0.001 |
| PD-L1 CPS | -1.100 | -2.538 | 0.339 | 0.131 |

Exploratory adjusted linear models for the association of preoperative log-transformed SII with tissue immune markers, adjusted for FIGO group and tumor size.

**Abbreviations:** CPS, combined positive score; SII, systemic immune-inflammation index.

**Supplementary Table S7. Sensitivity analyses assessing independence of the poor integrated immune-inflammatory phenotype from disease severity.**

| **Analysis** | **N** | **Events** | **HR (95% CI)** | ***P* value** | ***P* for interaction** |
| --- | --- | --- | --- | --- | --- |
| Unadjusted Cox | 612 | 119 | 3.29 (2.29, 4.72) | <0.001 | - |
| FIGO-stratified Cox | 612 | 119 | 2.43 (1.65, 3.58) | <0.001 | - |
| Subgroup Cox: IA-IB | 433 | 47 | 3.32 (1.81, 6.07) | <0.001 | 0.256 |
| Subgroup Cox: IIA-IIIC | 179 | 72 | 2.09 (1.27, 3.44) | 0.004 | 0.256 |
| Overlap-weighted Cox | 612 | 119 | 2.05 (1.41, 2.99) | <0.001 | - |
| Overlap-weighted doubly robust Cox | 612 | 119 | 2.46 (1.66, 3.65) | <0.001 | - |

Sensitivity analyses assessing whether the association between the poor integrated immune-inflammatory phenotype and recurrence-free survival persisted after accounting for disease severity. Overlap weighting was based on baseline severity-related clinicopathological variables.

**Abbreviations:** HR, hazard ratio.

**
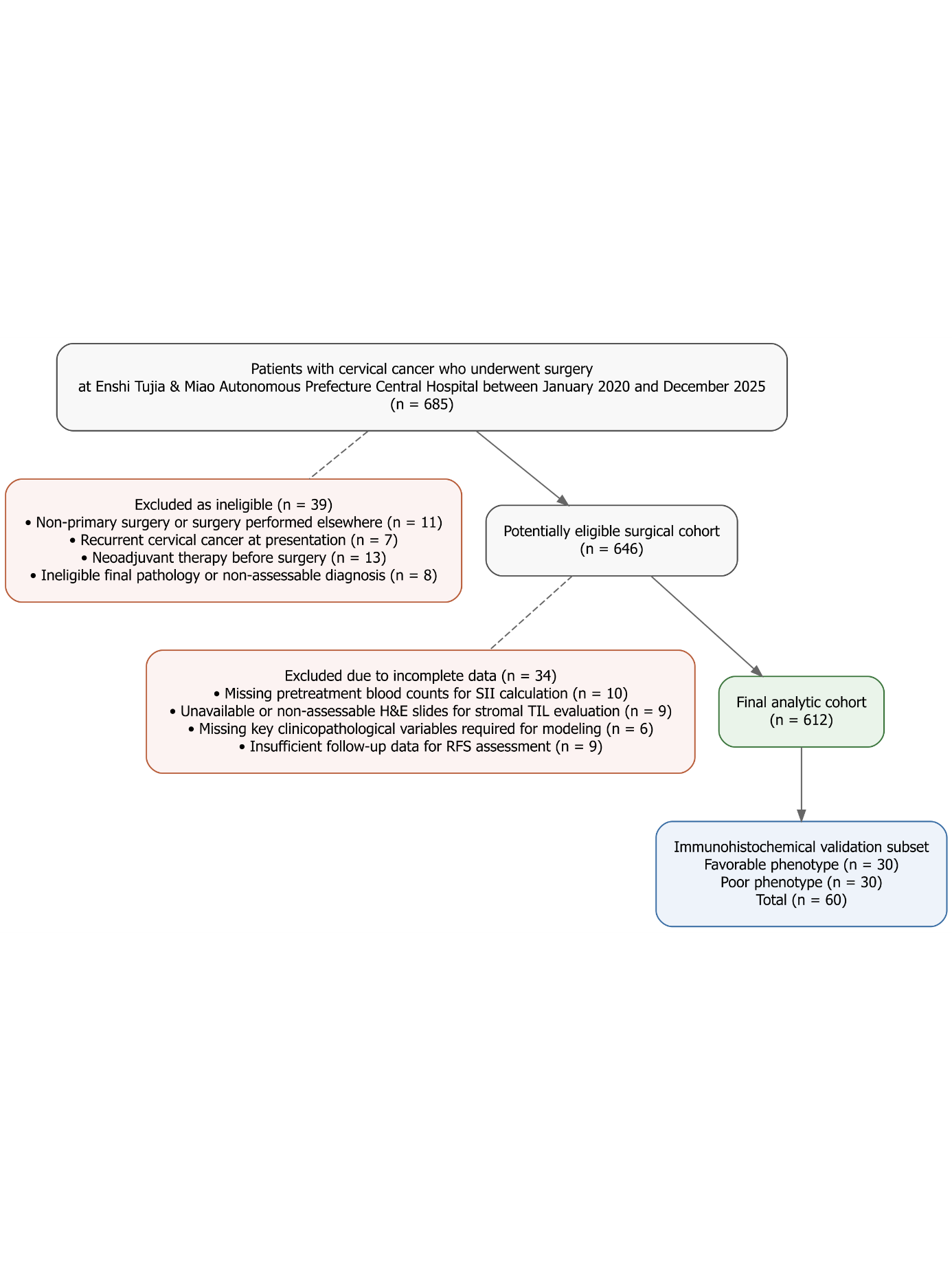
**

**Supplementary Figure S1.** **Flowchart of patient selection.** Flowchart showing cohort assembly, exclusion of ineligible cases, complete-case selection for the main prognostic analyses, and the subset included in immunohistochemical validation.

**
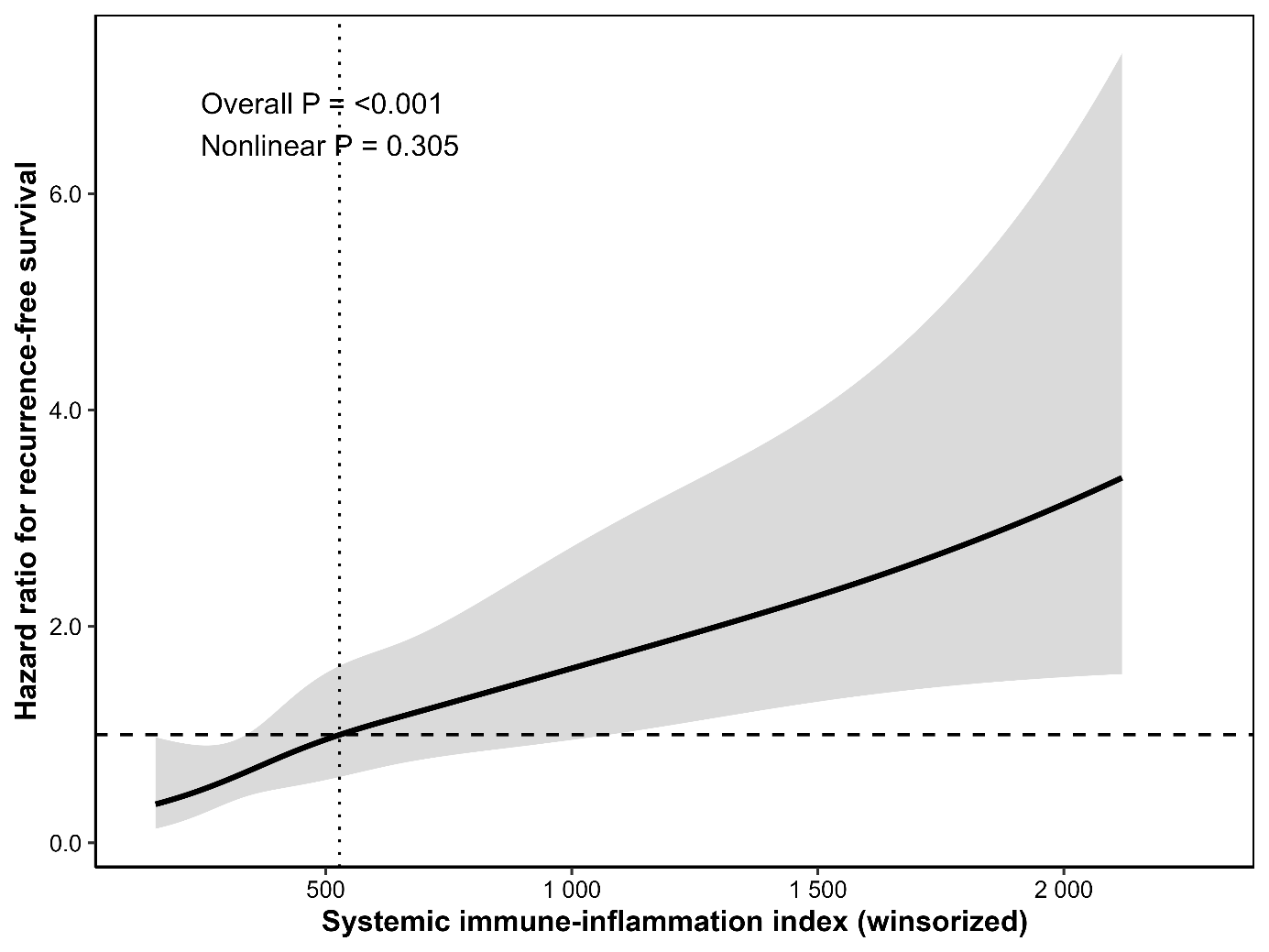
**

**Supplementary Figure S2. Restricted cubic spline analysis of the association between systemic immune-inflammation index and recurrence-free survival.**

**
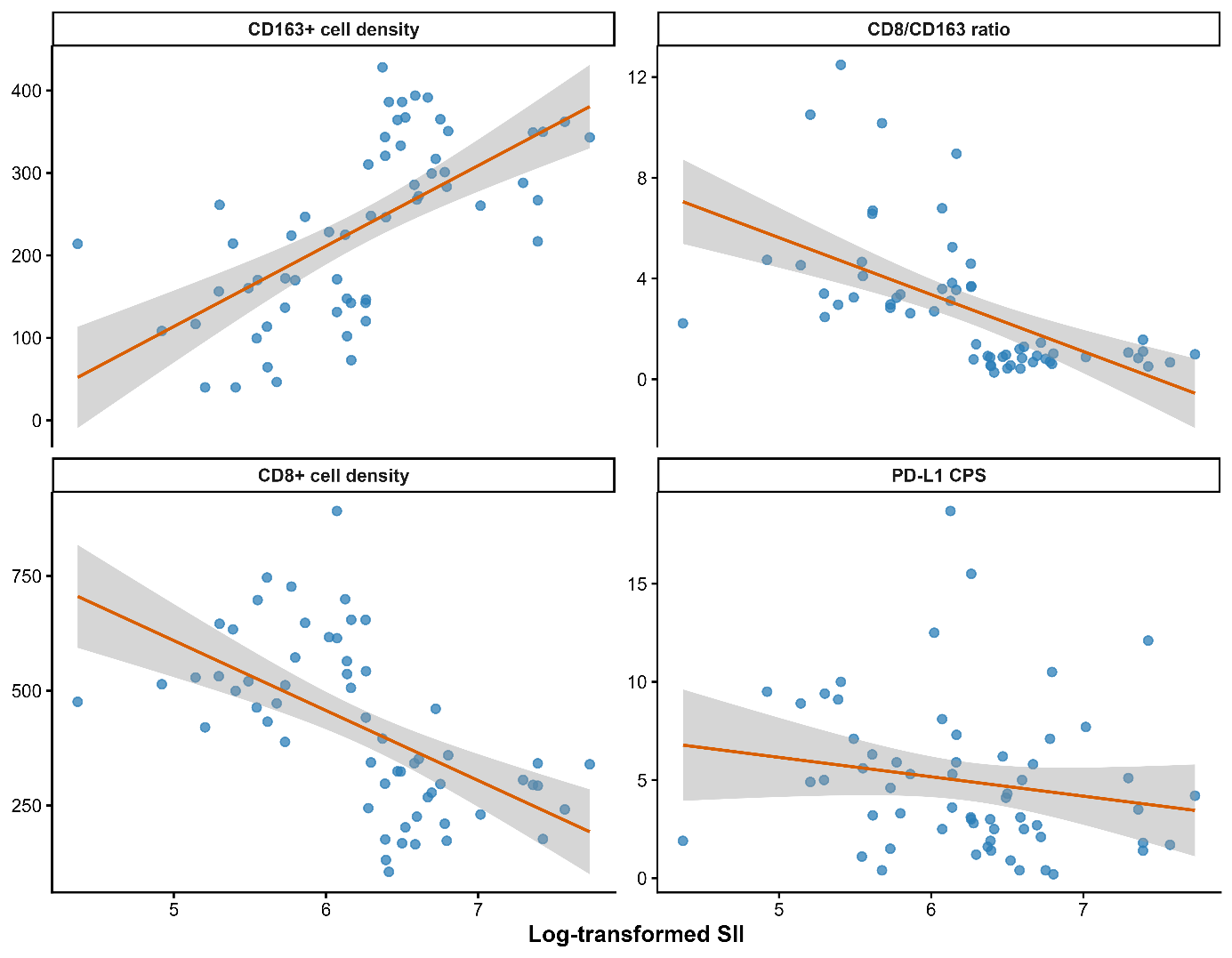
**

**Supplementary Figure S3. Exploratory associations between preoperative log-transformed SII and tissue immune markers in the immunohistochemical validation subset.** Scatterplots with fitted linear regression lines and 95% confidence bands showing the associations of log-transformed SII with CD163+ cell density, CD8/CD163 ratio, CD8+ cell density, and PD-L1 CPS.

**
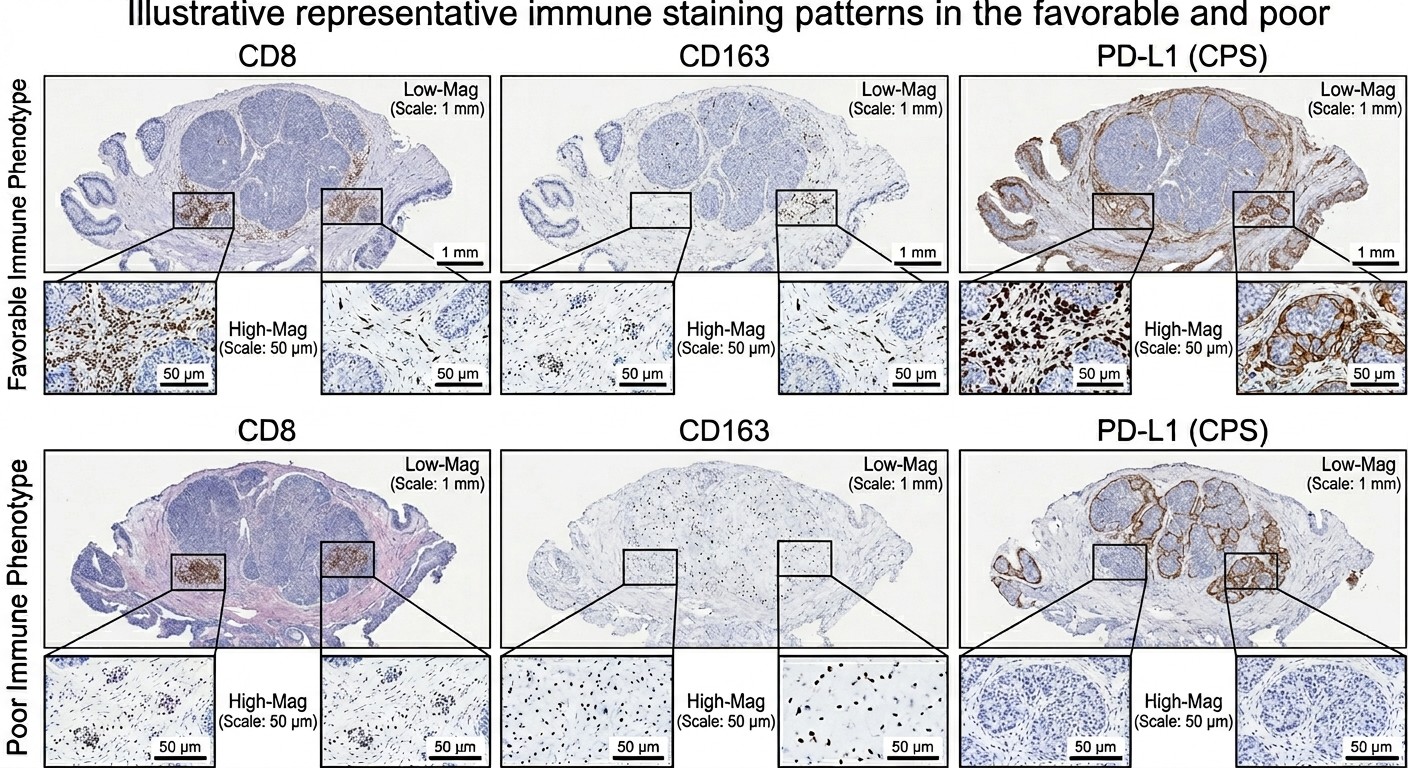
**

**Supplementary Figure S4. Representative immunohistochemical images in the favorable and poor integrated immune-inflammatory phenotype groups.** Low-magnification images show overall tumor structure, and boxed regions are shown at higher magnification. Representative staining patterns are shown for CD8, CD163, and PD-L1 (CPS). Scale bars: 1 mm and 50 μm.
